# Hidden blood loss and its risk factors after hip reconstruction in children

**DOI:** 10.1186/s13018-024-04861-2

**Published:** 2024-07-05

**Authors:** Jiafei Yang, Hua Jiang, Huajian Gu, Jun Du, Yingquan Zhuo, Kunfeng He, Song Yu

**Affiliations:** 1 School of Clinical Medicine, Guizhou Medical University, Guiyang, China; 2 Department of Pediatric Surgery, The Affiliated Hospital of Guizhou Medical University, Guizhou Medical University, Guiyang, China

**Keywords:** Hidden blood loss, Developmental dysplasia of the hip, Risk factors, Pemberton osteotomy, Children

## Abstract

**Objective:**

There were few reports in the literature regarding hidden blood loss following surgery for developmental dysplasia of the hip in children. This study aimed to evaluate the volume of hidden blood loss and its risk factors among children undergoing hip reconstruction for developmental dysplasia of the hip.

**Methods:**

A retrospective analysis of clinical data from 42 patients (58 hips), who underwent Pemberton and femoral osteotomies between March 2020 and March 2023, was conducted. Serial complete blood count assays were conducted on the day of admission and four days post-surgery. Preoperative and postoperative hematocrit levels were documented to calculate hidden blood loss utilizing the Gross formula. Pearson and Spearman correlation analyses, along with multivariable linear regression, were employed to ascertain associations between patient characteristics and hidden blood loss.

**Results:**

The mean hidden blood loss was recorded as 283.06 ± 271.05 mL, constituting 70.22% of the total blood loss. Multiple linear regression analysis identified weight and surgical duration as independent risk factors contributing to hidden blood loss.

**Conclusions:**

A relevant amount of postoperative hidden blood loss occurs after Pemberton osteotomy and femoral osteotomy for developmental dysplasia of the hip. Surgeons should be aware that patients who require blood transfusions and have longer surgical durations are at a higher risk of developing more hidden blood loss. Therefore, attention should be given to hidden blood loss to ensure patient safety during the perioperative period for those undergoing Pemberton and femoral osteotomies.

**Level of evidence:**

IV.

## Introduction

Developmental dysplasia of the hip (DDH) encompasses a broad spectrum of abnormal hip development during infancy and early childhood. This definition covers a wide range of severities, from mild acetabular dysplasia without hip dislocation to frank hip dislocation. Treatment of DDH typically entails open surgical intervention if nonsurgical approaches fail. The anterior surgical approach, facilitating broad capsular exposure, is generally preferred for children older than one year [1]. An acetabular osteotomy is typically performed after 18 months of age [2]. Femoral shortening constitutes a significant component of DDH’s surgical treatment, with excessive tension on the reduced hip potentially increasing the risk of proximal femoral growth disturbance, redislocation, and stiffness [3].

Since Sehat et al. [4] demonstrated that hidden blood loss (HBL) constituted 49% of the total blood loss after total hip replacement surgery, the significant impact of HBL on orthopedic procedures has been widely recognized by surgeons. Wu et al. [5] reported an HBL volume of 282 ± 163 ml following percutaneous kyphoplasty surgery. As a result, a growing body of research has underscored the prevalence of HBL in a range of orthopedic surgical procedures, including hip fracture surgery, total knee or hip arthroplasty, and spine surgery [6, 7]. Excessive HBL not only results in medical complications but also impacts postoperative rehabilitation and extends hospital stays, potentially affecting patient satisfaction [8]. Current research attributes the mechanism of HBL to factors such as blood stasis in the joint cavity and interstitial space, hemolysis, and postoperative bleeding [9]. HBL may result from the extravasation of blood from surgical wounds and fractures into the surrounding tissues. Although multiple studies [10] have documented the extent of blood loss following developmental dysplasia of the hip (DDH) surgery, no published research to date has explored the risk factors for HBL post-DDH procedure to our knowledge.

Surgeons typically provide estimated blood loss (EBL) to quantify the amount of blood lost during surgery. However, this estimation often underestimates the total functional blood loss as it fails to account for extravasated blood products that remain in the patient during and immediately after surgery—this unaccounted volume is termed “HBL”. Pemberton osteotomy (PO), increasingly utilized for treating DDH, primarily reports perioperative blood loss as intraoperative blood loss (IBL). Studies have shown that mean intraoperative blood loss for PO can range from 80 ml to 106 ml depending on the surgical approach [5, 6]. Despite this, Alassaf et al. [1] observed a blood transfusion rate of 11.8% following pelvic osteotomy for DDH, with no correlation to the IBL. This suggests that a significant portion of blood loss remains hidden. We hypothesize that HBL following PO for DDH may significantly elevate the risk of blood transfusion, highlighting the need for comprehensive blood loss assessment in these patients.

In this study, we conducted a retrospective analysis to evaluate the amount of HBL following DDH surgery and identified the influential factors contributing to HBL.

## Patients and methods

### Patients

Between May 2020 and May 2023, a total of 42 children (58 hips) underwent surgical procedures for hip capsulotomy and PFO at our institution (Pediatric Orthopedics unit of an academic hospital). For all children with bilateral DDH in our study, we opted for staged surgeries, performing a single-side surgery at a time, with the second surgery conducted six months later. This approach was chosen to minimize the surgical trauma and reduce the risk to the child’s life. Inclusion criteria included: confirmation of PFO post-DDH surgery, patients aged over 18 months and under 8 years, normal platelet count, prothrombin time, partial thromboplastin time, and international normalized ratio values, with no history of anticoagulant usage. Exclusion criteria were: patients aged over 8 years or under 18 months, prior hip surgery, cerebral palsy, tethered cord syndrome, or other neuromuscular diseases associated with hip dislocation, thromboembolic disease history, congenital or acquired coagulopathy, and hemorrhagic disease onset during hospitalization.

### Surgical procedure

All procedures were conducted by the same hip surgeon using a uniform technique, with each patient receiving identical anesthesia. A “Bikini” incision was utilized in all cases. The space between the tensor fascia lata and the sartorius muscle provided access to the rectus femoris, which was subsequently incised. A “T”-shaped incision was made in the joint capsule. The ligamentum teres was excised, and all fibrofatty tissue (pulvinar tissue) was removed from the true acetabulum. A second incision was made on the proximal thigh. The femur was exposed from the muscle’s posterior side, the periosteum separated, and a pre-bent, appropriately sized steel plate was selected in advance. The hip was reduced, and the overlap length was determined. Subsequently, a femoral shortening varus and/or rotational osteotomy was performed. The plate was affixed with screws. Using the center of the Y-shaped cartilage as the axis, the external iliac plate was cut 15 mm from the acetabulum’s upper edge, the distal end of the osteotomy reoriented forward, outward, and downward, and the gap filled with bone blocks. The joint capsule was then sutured, maintaining the hip joints in 30° flexion and abduction and 20–30° internal rotation. A drainage tube was inserted at the femoral osteotomy site.

### Postoperative management

Antibiotics were administered routinely for 48 hours post-operation to prevent infection. Strict postoperative fluid management protocols were implemented for all children. Post-surgery, it is recommended to initiate enteral feeding early. If early oral intake is impractical or insufficient, IV fluid support becomes crucial for maintaining normovolemia. The necessary IV fluid intake is calculated by deducting the oral intake from the child’s daily fluid requirements. Where parenteral fluid management is essential, children should receive isotonic solutions as maintenance fluids, adhering to the ‘4-2-1’ rule for fluid rates. The external fixation brace was applied, and subsequently removed after 6 weeks to commence functional exercise.

### Data collection

A complete blood count, including hematocrit (Hct) and hemoglobin (Hb), was conducted preoperatively and on the first four consecutive postoperative days. Intraoperative blood loss calculation involved weighing sponges used during each procedure and measuring the volume of blood in suction bottles through syringe pulls, subtracting the volume of lavage fluid utilized. By the second or third postoperative day, the patients would have achieved hemodynamic stability and any fluid shifts would be mostly complete [14]. The drainage tubes were removed 48 h after the procedures. All patients adhered to the updated blood transfusion guidelines recommending transfusion for Hb concentrations below 8 g/dL [15]. The demographic and medical information collected included the patient’s age, gender, weight, height, Body Mass Index (BMI), surgical site, surgical duration, pre- and postoperative acetabular index angle (AIA), amount of femoral shortening, blood loss, preoperative and postoperative Hct, Hb, prothrombin time (PT), activated partial thromboplastin time (APTT), and drainage volume. The volume of blood transfused was recorded as well. Using the Gross formula, hemoglobin(Hb) loss and total blood loss (TBL) were calculated. Pearson or Spearman correlation analysis was utilized to ascertain the relationship between hidden blood loss ratio to total blood volume and various parameters.

### Calculation of hidden blood loss

The patient’s blood volume (PBV) calculations were as follows [16]

Log PBV = 0.7891 log weight + 0.004132 height + 1.8117(infants and children younger than 2 years); log PBV (L) = 0.6459 log weight + 0.002743 height + 2.0324 for boys (boys aged 2 to 14 years and girls aged 2 to 6 years); log PBV (L) = 0.6412 log weight + 0.001270 height + 2.2169 for girls (girls aged 7 to 14 years).

The total blood loss(TBL) in the perioperative period was reflected by the reduction of Hct. It was calculated according to the method of Gross [17], using preoperative Hct(Hct_pre_), postoperative Hct(Hct_post)_ and PBV. The formula used was as follows: TBL(ml) = PBV(L) × (Hct_pre_ - Hct_post_)/ average Hct(Hct_ave_) × 1000, where Hct_pre_ referred to the preoperative Hct, Hctpost referred to the Hct on postoperative lowest day, and Hct_ave_ referred to the average of Hct_pre_ and Hct_post_. If an allogenic transfusion was performed, the TBL was smaller than expected because re-infusion artificially elevated the Hct. Therefore, the TBL was equal to the loss calculated from the Hct change plus the volume transfused [14]. The formula used was as follows: HBL = TBL-measured blood loss + blood infusion.

### Statistical analysis

SPSS 20.0 software was used to perform the statistical analysis(SPSS Inc, Chicago, IL). For continuous variables, mean, standard deviation, median, and quartiles were calculated, and for categorical variables, frequency was determined based on patient demographics and characteristics. Subsequently, Pearson and Spearman correlation analyses, along with multivariable linear regression analysis, were conducted to identify risk factors associated with HBL, including age, gender, height, weight, operation time, postoperative length of stay, and femoral shortening. The threshold for statistical significance was established at *p* < 0.05.

## Result

Between May 2020 and May 2023, 42 children (58 hips) were reviewed. The mean age of the children was 38.97 ± 23.52 months, mean height was 0.90 ± 0.15 m, and mean weight was 12.49 ± 4.00 kg. The mean surgical time was 164.23 ± 42.31 min. According to Tonnis’ classification, 17 hips were classified as type III and 41 hips as type IV. The mean postoperative change in AIA was 22.54 ± 6.83°. Additionally, the mean femoral height loss was 1.74 ± 0.67 cm, and the mean Hb change was 37.09 ± 14.08 g/L. Furthermore, the mean Hct change was 0.11 ± 0.04 (Table 1).

**Table 1:** Patient’s demographic information

Parameter (unit)	Mean ± SD
Total patients	42
Unilateral: Bilateral	26:16
Male: Female	8:34
Median age (month)	38.97 ± 23.52
Height (m)	0.90 ± 0.15
Weight (kg)	12.49 ± 4.00
BMI (kg/m^2^)	15.20 ± 1.98
Surgical site (Left/Right)	40/18
Tonnis’classification (Type III/Type IV)	17/41
Postoperative length of stay (day)	6.11 ± 2.73
The change of AIA^*^	22.54 ± 6.83
The height of femoral shortening (cm)	1.74 ± 0.67
PT^*^ (s)	12.64 ± 0.68
APTT^*^ (s)	40.99 ± 4.37
Surgical duration (minute)	164.23 ± 42.31
Change in Hb^*^ level (g/L)	37.09 ± 14.08
Change in Hct^*^ level	0.11 ± 0.04

Normal distribution data are presented as mean ± SD, non-normal distribution data are presented as mean

*BMI = body mass index, AIA = acetabular index angle, PT = prothrombin time, APTT = activated partial thromboplastin time, Hb = haemoglobin, Hct = hematocrit

**Fig. 1:**
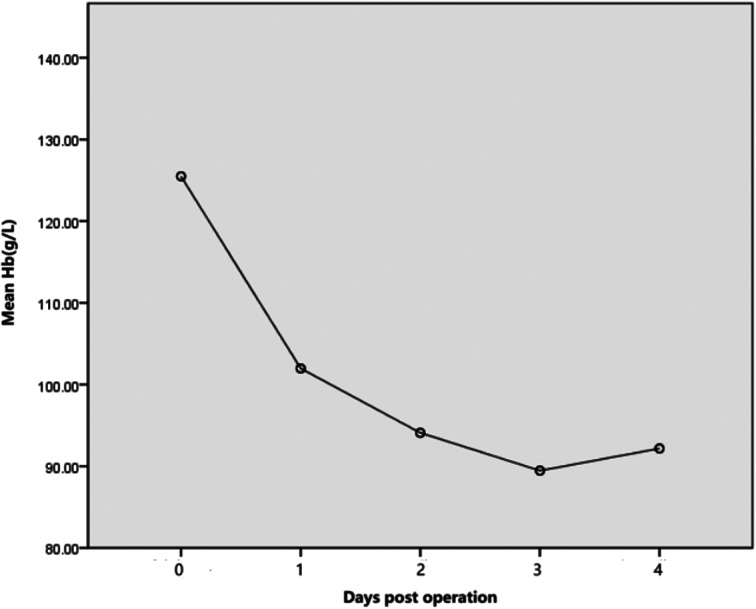
Postoperative trend in mean hemoglobin dynamic variation

Table 2 displays data on intraoperative bleeding, Hb level loss, calculated blood loss (HBL and TBL), transfusion rate, and HBL percentage. The mean TBL amounted to 403.67 ± 351.99 mL, and the mean HBL was 283.06 ± 271.05 mL, constituting 70.22 ± 23.16% of TBL. Figure 1 illustrates the mean serial Hb levels and their dynamic trends over the first 4 days post-surgery. Fourteen children, representing 24.13% of the total, received blood transfusions Table 3 shows the ratio of hidden blood loss to total blood volume across different body weight categories.

**Table 2:** Perioperative Hb values, blood loss, and transfusion data

Variable	Mean ± SD	Median (IQR)
Hb value (g/L)		
Admission day	125.49 ± 9.04	125(11)
Postoperative day 1	101.97 ± 12.35	102(16)
Postoperative day 2	94.09 ± 9.61	97(10)
Postoperative day 3	89.46 ± 12.19	91(10)
Postoperative day 4	92.17 ± 12.40	93(10)
Intraoperative observed blood loss (mL)	110.61 ± 80.94	93(87)
Perioperative hidden blood loss (mL)	283.06 ± 271.05	169.08(178.18)
Total perioperative blood loss (mL)	403.67 ± 351.99	289.161(259.33)
Transfusion rate, n (%)	14(24.13)	
Percentage of hidden blood loss in total(%)	70.22 ± 23.16	

Hb = haemoglobin

**Table 3:** The ratio of hidden blood loss to total blood volume in different body weight

Weight(Kg)	Number of cases	Mean HBL% ± SD
≥ 5 and <10	10	18.20 ± 8.75
≥ 10 and <15	34	25.29 ± 5.26
≥ 15 and <20	9	27.42 ± 9.01
≥ 20 and <25	5	40.48 ± 5.85

The analysis revealed that age, height, weight, BMI, surgical duration, the change of AIA, the height of femoral shortening and gender had a significant correlation with the ratio (*P* < 0.05) (Table 4 and Table 5). Further, multivariable linear regression analysis identified weight and surgical duration as independent risk factors for the percentage of hidden blood loss (Table 6), pinpointing weight (95% confidence interval = [0.304, 3.465], *P* = 0.021], surgical duration (95% confidence interval = [0.004, 0.302], *P* = 0.016) as the independent risk factors for postoperative HBL.

**Table 4:** Multivariable linear regression analysis on risk factors of hidden blood loss percentage-wise (percentage of total blood volume)

Parameter	Sig, (2-tailed)	p value
Age	0.5	0.002
Height	**0.479**	0.004
Weight	0.606	0.001
BMI	**-0.346**	0.042
Surgical duration	0.637	p<0.001
The change of AIA	**0.345**	0.042
The height of femoral shortening	**0.393**	0.02

BMI=body mass index; AIA=acetabular index angle

**Table 5:** Result of Spearman correlation analysis for hidden blood loss percentage-wise(percentage of total blood volume)

parameter	Sig, (2-tailed)	*p* value
**Gender**	**-0.348**	**0.041**
Surgical site	-0.177	0.308

**Table 6:** Multivariable linear regression analysis on risk factors of hidden blood loss percentage-wise(percentage of total blood volume)

	B	95%CI		*P*	
		Lower	Upper		
Age	-0.062	-8.47968	8.35568	0.472	
Gender	-0.035	-0.575787	0.505787	0.605	
Height	-0.028	-0.431755	0.375755	0.538	
**Weight**	**1.885**	**0.304238**	**3.465762**	**0.021**	
BMI	-0.205	-4.360006	3.950006	0.952	
**Surgical duration**	**0.153**	**0.003733**	**0.302267**	**0.016**	
The change of AIA	0.185	-3.404749	3.774749	0.895	
The height of femoral shortening	-0.039	-0.667879	0.589879	0.665	

## Discussion

This study uncovered a significant prevalence of HBL following DDH surgery in children, exceeding initial expectations. Our findings indicate that both HBL and the average Hb loss were significantly greater than reported in earlier studies concerning HBL ratios. The IBL findings were akin to those reported by Bulut in a study on PFO surgery [18]. It is important to note that 14 cases (24.13%) required blood transfusion during the perioperative period. Recently, surgeons have placed greater emphasis on perioperative bleeding in children, yet research specifically targeting HBL in this demographic remains scarce. Femoral and pelvic osteotomy in PFO surgery lead to two osteotomy ends and wounds, causing substantial blood flow into the tissue space. These findings are consistent with prior research indicating that longer incisions and more extensive soft tissue dissection result in increased HBL [9, 14]. Post-surgery, children frequently suffer from severe anemia, extending beyond the first day after the operation. Our research indicates that the lowest hemoglobin levels are not reached on the first day after surgery. Daily pre- and postoperative blood tests (Table 2; Figure 1) reveal that Hb loss reaches its nadir between the second and fourth day post-surgery. This aligns with Ju et al.‘s study [19], which found patients to be hemodynamically stable between the second and fourth day post-operation, with most fluid shifts concluded by this time. However, children endure greater blood loss and encounter more challenges in blood management compared to adults. At present, definitive guidelines for blood management in children with HBL are lacking, due to the limited number of studies with small sample sizes exploring effective strategies. Additionally, it is important to note that 14 cases of anemic patients were observed postoperatively, accounting for 24.13% of all cases. Alassaf et al. [20] analyzed 137 patients undergoing pelvic osteotomy and femoral shortening, finding a 27.41% transfusion rate, aligning with our results. Blood transfusions are linked to longer hospital stays and increased complication rates, underscoring the importance of effective patient evaluation during the perioperative period.

Numerous studies on HBL in adults have identified various independent risk factors, including gender, hypertension, diabetes, and heart disease [20, 21]. Previous research has underscored age as a significant factor influencing perioperative HBL [22]. The results showed that weight and surgical duration are positive independent risk factors for HBL. This study presents a novel finding: body weight may be an independent risk factor for elevated hidden blood loss in children. Currently, no studies report on the relationship between body weight and HBL in children. However, an established linear relationship exists between body weight and age in children. Previous studies have demonstrated that older age is associated with a higher risk of perioperative allogeneic blood transfusion [21]. Regarding age, in patients with femoral intertrochanteric fractures, it was found that HBL was significantly higher in those over 60 years compared to those under 60 [22]. Although our study did not reveal a significant linear relationship between age and HBL through multiple linear regression analysis, the small sample size may be the primary reason. We hypothesize that older children possess more interstitial spaces, potentially leading to increased HBL.

Surgical duration as a risk factor for requiring blood transfusion and found a positive correlation with perioperative bleeding in PFO [23],. Our findings align with earlier research [19, 24]. In this study, the average HBL reached approximately 283 ml, constituting around 70% of TBL for surgeries exceeding 3 h. Patients whose surgeries exceeded 3 h accounted for 78% of all blood transfusions in this study. As operative time increased, so did the volume of blood oozing in the surgical field, with the surgical wound remaining open and unable to form a closed pressurized cavity, this resulted in more opportunities for exposed surfaces to bleed and the continuous exposure of the two osteotomy surfaces, leading to increased oozing blood volume and, consequently, enhanced local blood flow into the tissue space. This oozing likely accounts for the continuous blood loss observed up to the closure of the muscle fascia, including HBL [23, 25]. These factors illustrate the relationship between HBL and surgical duration.

In this study, we identified weight and surgical duration as significant independent risk factors for HBL. Previous studies have attributed the mechanism of HBL to factors such as blood stasis in the joint cavity and interstitial space, hemolysis, and postoperative bleeding. These findings underscore the importance of monitoring and managing these risk factors to minimize HBL and improve patient outcomes following DDH surgery. Surgeons are advised to assess anticipated blood loss and determine if it surpasses visible loss for each patient. This assessment necessitates evaluating the patient’s erythrocyte stock. If anticipated blood loss exceeds allowable limits, tranexamic acid may reduce HBL and post-surgery transfusion rates [26]. Perioperative blood transfusions can extend patient recovery times and delay hospital discharge.

This study had several limitations that need acknowledgment. Firstly, we conducted a retrospective analysis, and the samples in our study were relatively small, which cannot exclude the effect of other potential factors on the results. Secondly, the study may have underestimated the effects of hemodilution from perioperative fluid infusion. Thirdly, the assessment of hemodynamic stability was based on HBL estimates on day 3 or 4 postoperatively, although other reports indicate the lowest HBL occurs between days 4 and 7 [10]. Due to these limitations, further high-quality observational and basic experimental studies are needed to explore new risk factors for HBL in patients undergoing PFO.

## Conclusion

Consequently, the study concluded that patients undergoing PFO incur a significant amount of HBL. Factors including weight and surgical duration significantly impact HBL in surgical patients. This study is the first to identify weight as a critical factor in predicting HBL in children. Incorporating these parameters into a predictive model could potentially reduce the need for blood transfusions, lower costs, and shorten hospital stays during the perioperative period.

## Data Availability

Author Jiafei Yang can provide original data upon reasonable request. His email is 1211355517@qq.com.

## Abbreviations

HBL: Hidden blood loss
PFO: Pemberton osteotomy and femoral osteotomy
DDH: Developmental Dysplasia of the Hip
Hct: Hematocrit
Hb: Hemoglobin
BMI: Body Mass Index
AIA: Acetabular index angle
PT: Prothrombin time
APTT: Partial thromboplastin time
PBV: The patient’s blood volume
Hct_pre_: Preoperative Hct
Hct_post_: Postoperative Hct
Hct_ave_: Average Hct

## References

[1] Alassaf N, Reitsma JB (2019). Development of a prediction model for allogenic blood transfusion in children undergoing surgery for developmental dysplasia of the hip[J]. Technol Health Care.

[2] Thomas SR, Wedge JH, Salter RB (2007). Outcome at forty-five years after open reduction and innominate osteotomy for late-presenting developmental dislocation of the hip[J]. JBJS.

[3] Alassaf N (2018). Predictors of femoral shortening for pediatric developmental hip dysplasia surgery: an observational study in 435 patients[J]. Patient Saf Surg.

[4] Sehat KR, Evans R, Newman JH. How much blood is really lost in total knee arthroplasty? Correct blood loss management should take hidden loss into account[J]. Knee. 2000 July;1(3):151–5. 10.1016/s0968-0160(00)00047-8 10927208

[5] Wu Y, Zhang H, Zheng W, Feng Z, Chen Z, Lin Y (2017). Hidden blood loss and the influential factors after percutaneous kyphoplasty surgery[J]. Eur Spine J.

[6] Guo W, Wang J, Zhang W, Wang W, Xu D, Luo P (2018). Hidden blood loss and its risk factors after hip hemiarthroplasty for displaced femoral neck fractures: a cross-sectional study[J]. Clin Interv Aging.

[7] Miao K, Ni S, Zhou X, Xu N, Sun R, Zhuang C (2015). Hidden blood loss and its influential factors after total hip arthroplasty[J]. J Orthop Surg Res.

[8] Liu X, Zhang X, Chen Y, Wang Q, Jiang Y, Zeng B (2011). Hidden blood loss after total hip arthroplasty[J]. J Arthroplast.

[9] Li S (2022). The hidden blood loss and its factors in patients undergoing minimally invasive knee arthroscopy[J]. Front Surg.

[10] Adam C, Adler, Lisa AH, Hensch BE, Bryant A, Chandrakantan H-Y, Nguyen BH, Nathanson (2022). Factors affecting need for blood transfusion in paediatric patients undergoing open surgery for hip dysplasia[J]. Vox Sang.

[4] Badrinath R, Bomar JD, Wenger DR, Mubarak SJ, Upasani VV (2019). Comparing the Pemberton osteotomy and modified San Diego acetabuloplasty in developmental dysplasia of the hip[J]. J Child Orthop.

[5] Yilar S, Zencirli K, Köse M, Ezirmik N (2020). Comparison of total cost and outcomes between single-stage open reduction and Pemberton periacetabular osteotomy operation and two separate consecutive operations in treatment of bilateral developmental hip dysplasia in children at walking age[J]. J Pediatr Orthop B.

[6] Su Y, Nan G. Modified Pemberton Pelvic Osteotomy through Inner Ilium Approach for Treatment of Developmental Dysplasia of the hip in Children[J]. Indian J Orthop 2022 June 27; 56(9): 1625–33. 10.1007/s43465-022-00676-7 PMC9385895 36052389

[14] Jiang C, Chen TH, Chen ZX, Sun ZM, Zhang H, Wu YS. Hidden blood loss and its possible risk factors in cervical open-door laminoplasty[J]. J Int Med Res 2019 June 24; 47(8): 3656–62. 10.1177/0300060519856987 PMC6726792 31234677

[15] Carson JL, Guyatt G, Heddle NM, Grossman BJ, Cohn CSC, Funk MK (2016). Clinical practice guidelines from the AABB: red blood cell transfusion thresholds and storage[J]. JAMA.

[16] Linderkamp O, Versmold HT, Riegel KP (1977). Estimation and prediction of blood volume in infants and children. Eur J Pediatr.

[17] Gross JB (1983). Estimating allowable blood loss: corrected for dilution[J]. J Am Soc Anesthesiologists.

[18] Bulut M, Azboy I, Ozkul E, Lokman (2021). Comparison of iliac and femoral autograft practices in Pemberton pelvic osteotomy[J]. J Pediatr Orthop.

[19] Ju H, Hart RA (2016). Hidden blood loss in anterior lumbar interbody fusion (ALIF) surgery[J].

[20] Zhixiang G, Cong X, Hongtao Y, Xiangyu M (2021). Statistical decision tree model analysis on hidden blood loss in the perioperative period of thoracolumbar burst fracture accompanied with neurological deficiency. Chin J Tissue Eng Res.

[21] Michael S, Jürgen W, Sarah-Jayne E, Irene M, Jasmin R, Thomas G (2021). Is there a hidden blood loss in orthognathic surgery and should it be considered? Results of a prospective cohort study. J Craniomaxillofac Surg.

[22] Cui H, Chen K, Lv S, et al. An analysis of perioperative hidden blood loss in femoral intertrochanteric fractures: bone density is an important influencing factor[J]. BMC Musculoskelet Disord. 2021;22(1). 10.1186/s12891-020-03922-x 10.1186/s12891-020-03922-x PMC7784311 33397328

[23] Sherrod BA, Baker DK, Gilbert SR (2018). Blood transfusion incidence, risk factors, and associated complications in surgical treatment of hip dysplasia[J]. J Pediatr Orthop.

[24] Wang JQ, Huang XJ, Guo WJ, Zhao YM, Luo P. Hidden blood loss and the influential factors after intramedullary nail fixation of extra-articular tibial fractures-a retrospective cohort study[J]. Injury. 2020 June;51(6):1382–6. 10.1016/j.injury.2020.04.025 32327232

[25] Lei F, Li Z, He W, Tian X, Zheng L, Kang J, Feng D. Hidden blood loss and the risk factors after posterior lumbar fusion surgery: a retrospective study[J]. Medicine May 8, 2020, 99(19). 10.1097/MD.0000000000020103 PMC7440351 32384484

[26] Xu D, Chen X, Li Z, Ren Z, Zhuang Q, Li S. Tranexamic acid reduce hidden blood loss in posterior lumbar interbody fusion (PLIF) surgery[J]. Med May 13, 2020, 99(11). 10.1097/MD.0000000000019552 PMC7440332 32176112

